# Identification of distinct gene co‐expression modules and specific hub genes in skin lesions of atopic dermatitis and psoriasis by WGCNA

**DOI:** 10.1002/2211-5463.13686

**Published:** 2023-08-12

**Authors:** Yu Sheng, Jing Liu, Shuyun Zheng

**Affiliations:** ^1^ Department of Dermatology The First Affiliated Hospital of Harbin Medical University China

**Keywords:** atopic dermatitis, psoriasis, WGCNA

## Abstract

Atopic dermatitis (AD) and psoriasis are among the most common chronic inflammatory skin diseases. Although AD and psoriasis are distinguished using clinical criteria, the lesions of these two diseases are sometimes highly similar, making diagnosis difficult. In addition, the mechanisms underlying these two diseases are not fully clear. Here, we aimed to identify potential genes and regulatory mechanisms in AD and psoriasis patients to aid in the diagnosis and treatment of AD and psoriasis. The GSE121212 dataset was obtained from the NCBI Gene Expression Omnibus database and weighted gene co‐expression network analysis (WGCNA) was applied. The functions of genes in modules of interest were determined using Gene Ontology enrichment analysis and Kyoto Encyclopedia of Genes and Genomes analysis with the ggplot2 package of r. The hub genes were obtained using the Search Tool for the Retrieval of Interacting Genes database and then visualized using cytoscape. The MEgreen and MEbrown modules were identified to associate with AD and psoriasis, respectively, and the biological functions and pathways of genes in clinically significant modules were detected and analyzed. Hub genes in these two modules and details on potential protein interactions were also revealed. The genes and modules identified by WGCNA might contribute to our understanding of the molecular mechanisms of AD and psoriasis and aid in their diagnosis and treatment.

## Graphical abstract

Atopic dermatitis (AD) and psoriasis are both important chronic inflammatory skin diseases. We performed weighted gene co‐expression network analysis (WGCNA) to construct a co‐expression network of AD and psoriasis in the GSE121212 dataset to predict clusters of genes involved in the pathogenesis of AD and psoriasis, and found potential hubgenes that might contribute to the understanding of the molecular mechanism of AD and psoriasis.

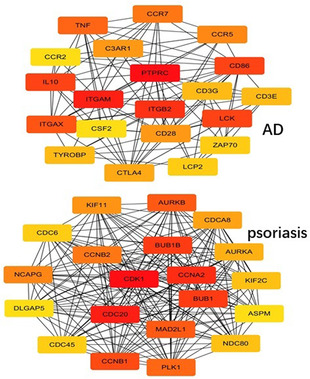

**Abbreviations**
AD: atopic dermatitis
CAMS: cell adhesion molecules
DAVID: Database for Annotation, Visualization and Integrated Discovery
GEO: Gene Expression Omnibus
GO: Gene Ontology
HTLV‐1: human T cell leukemia virus type 1
KEGG: Kyoto Encyclopedia of Genes and Genomes
PPI: protein–protein interaction
STRING: Search Tool for the Retrieval of Interacting Genes
TOM: topological overlap matrix
WGCNA: weighted gene co‐expression network analysis

Atopic dermatitis (AD) and psoriasis are two types of the most common chronic inflammatory skin diseases that carry a major health burden, although the exact mechanism of these two disease is not fully clear. Weighted gene co‐expression network analysis (WGCNA) is a systematic biological method used to describe gene association patterns among different samples [1]. It can be used to identify highly collaborative gene sets, as well as to identify candidate biomarkers or therapeutic targets based on correlation between gene sets and phenotypes [1]. In addition, it can be used to compare genes and help to study the interaction between genes in different modules [2]. Accordingly, it is of interest to further explore the mechanism of AD and psoriasis using this method.

Although AD and psoriasis are distinguished using clinical criteria, sometimes they have very similar lesions that make diagnosis difficult. At present, the clinical diagnosis of psoriasis is mainly based on the characteristics of skin lesions, the site of disease and histopathological changes. In the daily clinical practice for an expert dermatologist, it is easy distinguish AD and psoriasis. In some rare cases, histology is needed, but the atypical cases of skin lesions are also difficult to be diagnosed by histopathology [3]. In addition, the specificity and sensitivity of laboratory routine examination indicators in the diagnosis of psoriasis is low, and the clinical value and significance are limited. Therefore, it is necessary to establish a highly specific diagnostic method for psoriasis. Biomarkers are indicators that can objectively measure and evaluate the normal biological process, pathological process or response to drug intervention [4]. They are of great value in the development of new drugs, medical diagnosis and clinical research, and help researchers to propose more effective diagnosis and treatment methods. At present, the lack of specific biomarkers for the diagnosis of psoriasis is one of the important defects in the diagnosis and treatment of psoriasis.

Previous studies have made comparisons between AD and healthy people or between psoriasis and healthy people, but few studies have compared AD and psoriasis. In the present study, we used the WGCNA to identify AD specific genes and psoriasis specific genes, providing insights into their molecular mechanisms and identifying potentially biomarkers for differential diagnosis. WGCNA was constructed on the basis of data from GSE121212, which included 27 AD patients, 28 psoriasis patients and 38 healthy controls. Two significant gene modules associated with AD and psoriasis, respectively, were identified, and the biological functions and pathways of genes in clinical significant modules were detected and analyzed. Hub genes in these two modules and information on their protein interactions were also revealed. We postulated that these genes and modules might be potential biomarkers for the diagnosis and treatment of AD and psoriasis and might contribute to our understanding of the molecular mechanisms of their pathogenesis.

## Materials and methods

### Data information

Raw gene expression profile and clinical data were obtained from NCBI Gene Expression Omnibus (GEO) database (http://www.ncbi.nlm.nih.gov/geo). The GSE121212 expression data set contains 147 transcriptome sequencing results, and consists of 27 AD patients, 28 psoriasis patients and 38 healthy controls. The platform is GPL16791 lllumina HiSeq 2500 (*Homo sapiens*).

### WGCNA construction and identification of clinical significant modules

The WGCNA package of r language (R Foundation, Vienna, Austria) was used to identify clinical traits‐related modules in GSE121212. The soft threshold was selected to perform clustering analysis on gene expression, and the possible gene modules associated with disease were screened. The adjacency matrix was transformed into topological overlap matrix (TOM). According to the TOM‐based dissimilarity measure, the genes were divided into different gene modules. We calculated the correlation between gene modules and clinical trait to identify the relevant modules. The gene modules with the strongest correlation with clinical feature were selected as clinical significant modules.

### Function enrichment analysis

To further clarify the function of genes in interest modules, we performed Gene Ontology (GO) (http://geneontology.org) enrichment analysis and Kyoto Encyclopedia of Genes and Genomes (KEGG) (https://www.genome.jp/kegg) analysis for modules using the Database for Annotation, Visualization, and Integrated Discovery (DAVID) [5], and the ggplot2 package of r language is used for visual analysis.

### Protein–protein interaction network construction and hub genes identification

We used the Search Tool for the Retrieval of Interacting Genes (STRING) database (https://string‐db.org) to build protein–protein interaction (PPI) networks and then visualized using cytoscape (https://cytoscape.org). Hub genes that are highly interconnected with nodes in a module have been considered as functionally significant. We chose hub genes ranked top 20 degree in PPI networks as the candidate genes.

## Results

### Data set quality assessment

**Fig. 1:**
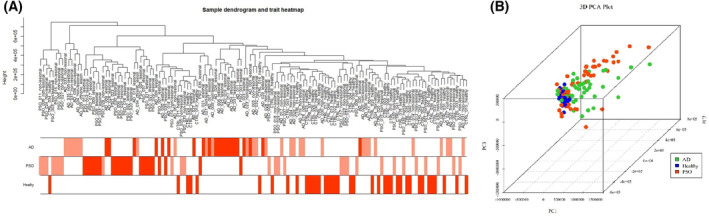
(A) The clustering was based on the expression data of GSE121212, which contained 27 AD patients, 28 psoriasis patients and 38 healthy controls. (B) Principal component analysis showed variability between different data. PSO, psoriasis.

Cluster analysis was carried out on 147 transcriptome data, and 10 groups of data with abnormal gene expression data were removed from subsequent analysis in GSE121212. A preliminary overview of the relationship between gene expression and clinical traits was conducted using cluster and heat maps (Fig. 1A). Principal component analysis showed variability between different data (Fig. 1B).

### WGCNA construction and identification of clinical significant modules

**Fig. 2:**
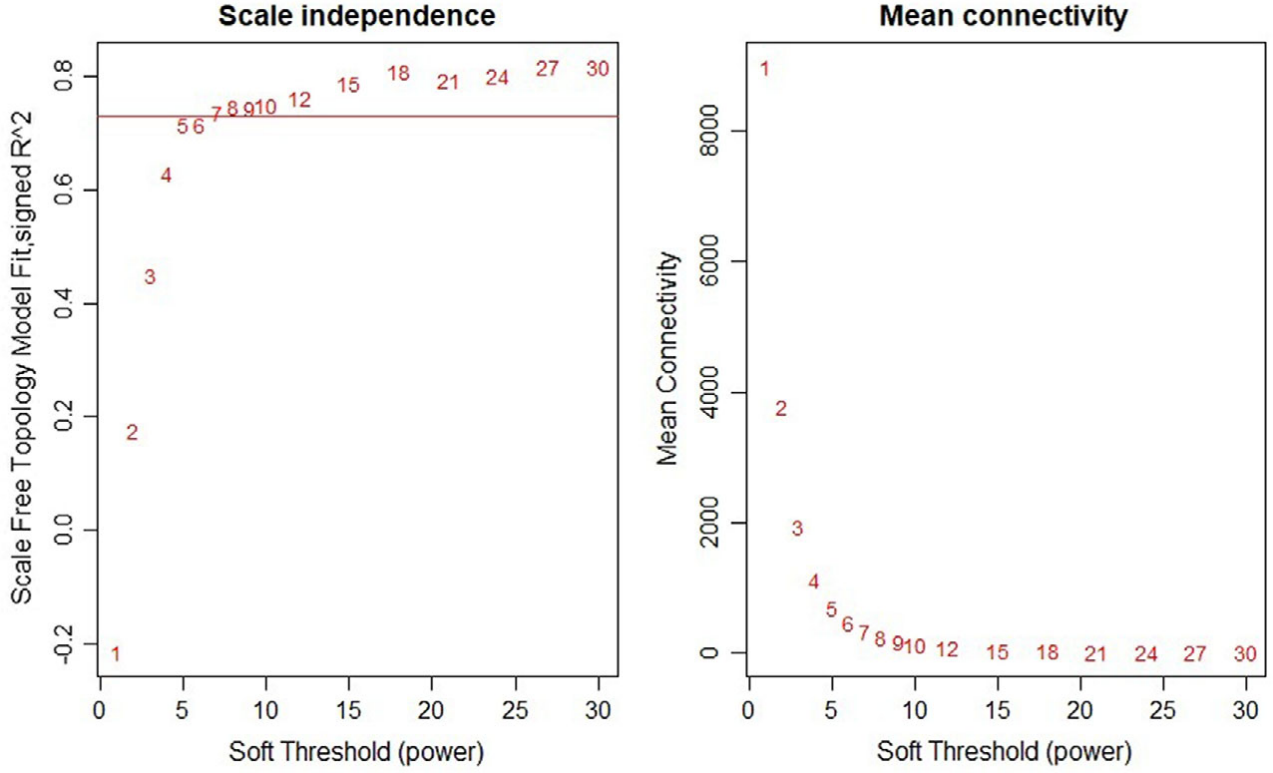
Analysis of the scale‐free fit index for various soft‐thresholding powers and the mean connectivity for various soft‐thresholding powers.

**Fig. 3:**
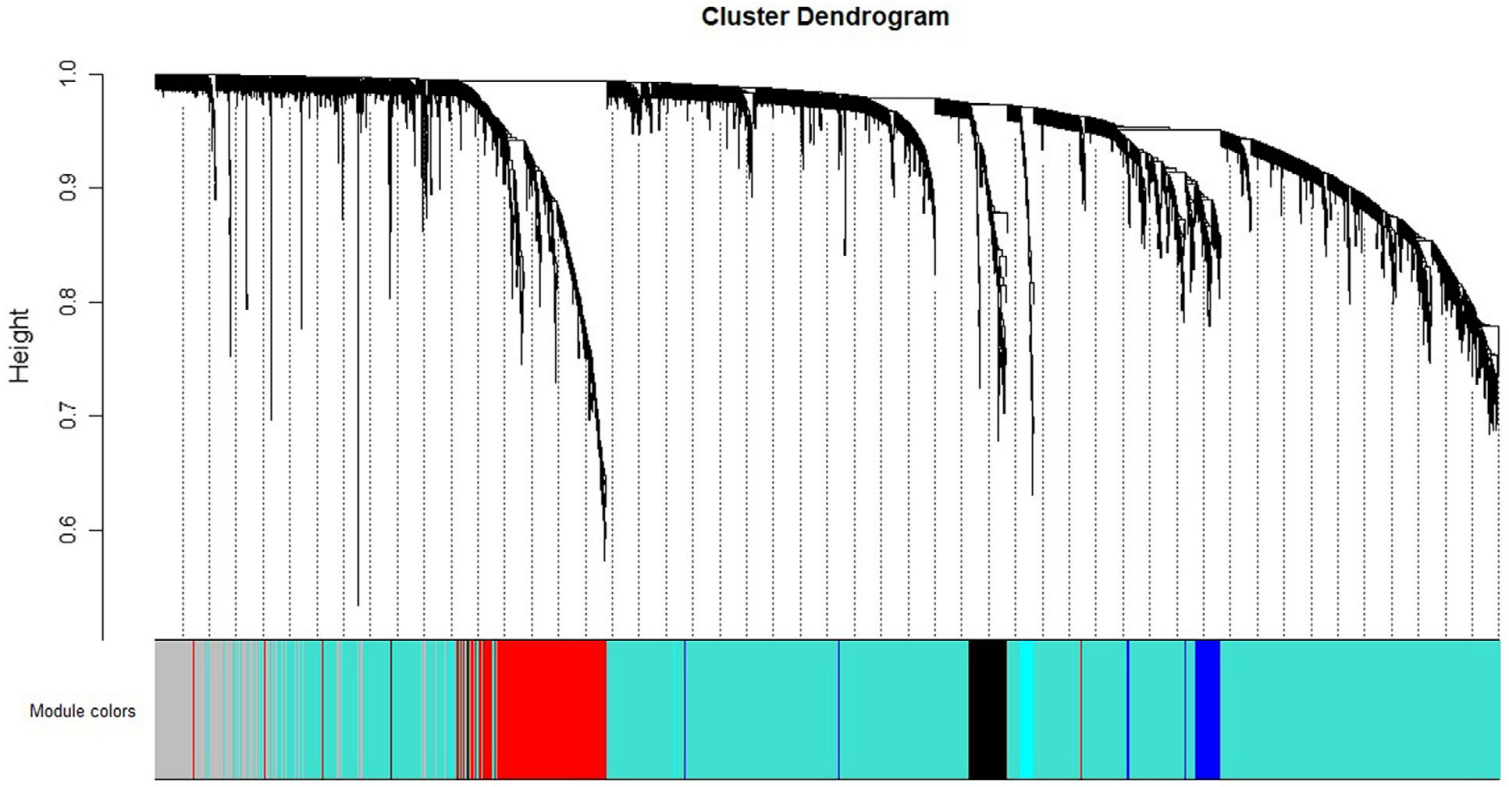
The cluster dendrogram of genes in GSE121212. Each branch represents one gene, and every color below represents one co‐expression module.

**Fig. 4:**
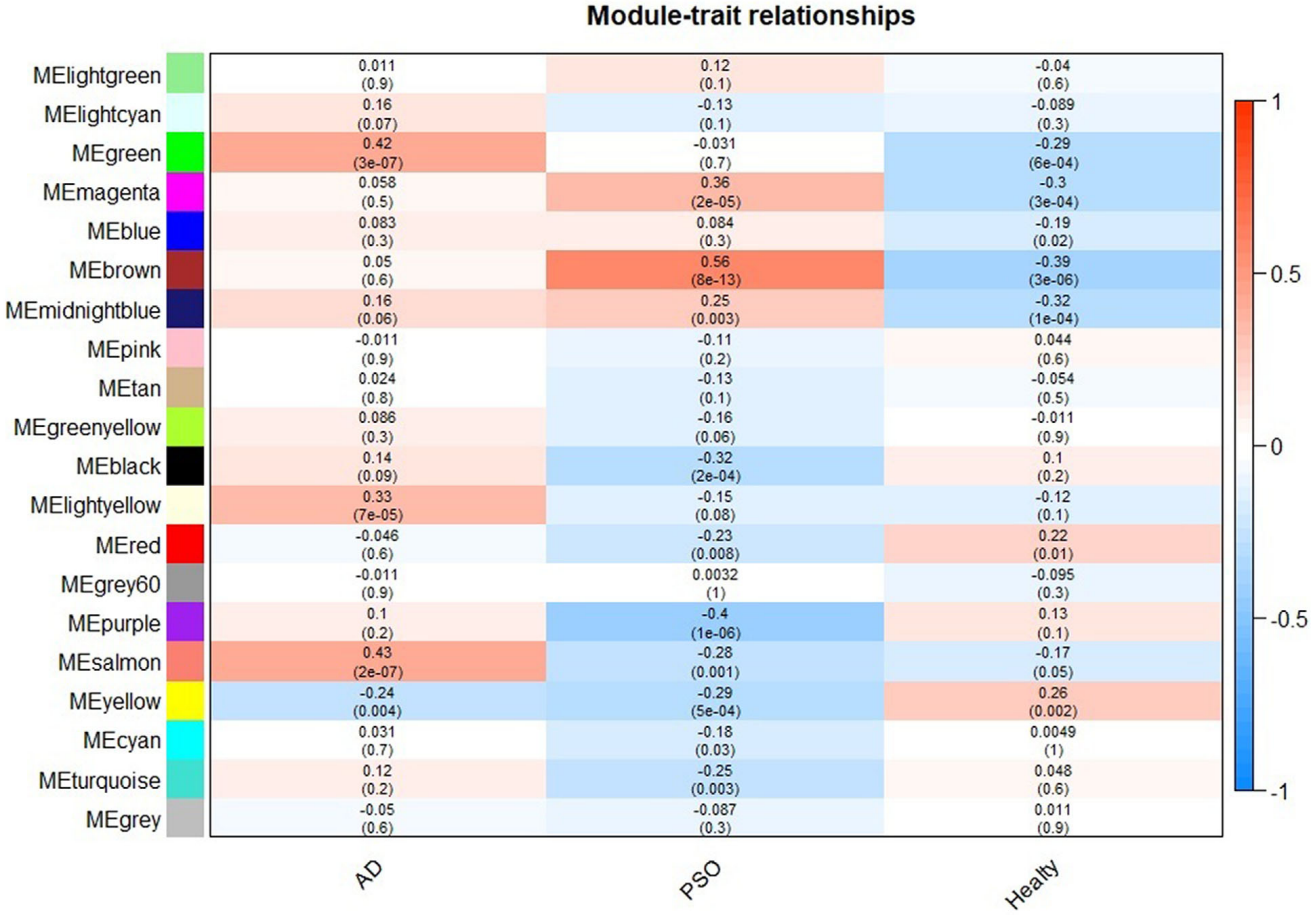
Heatmap of the correlation between module eigengenes and the disease. The MEgreen identified was the most significantly related module to AD and the MEbrown module was the most relevant module to psoriasis.

**Fig. 5:**
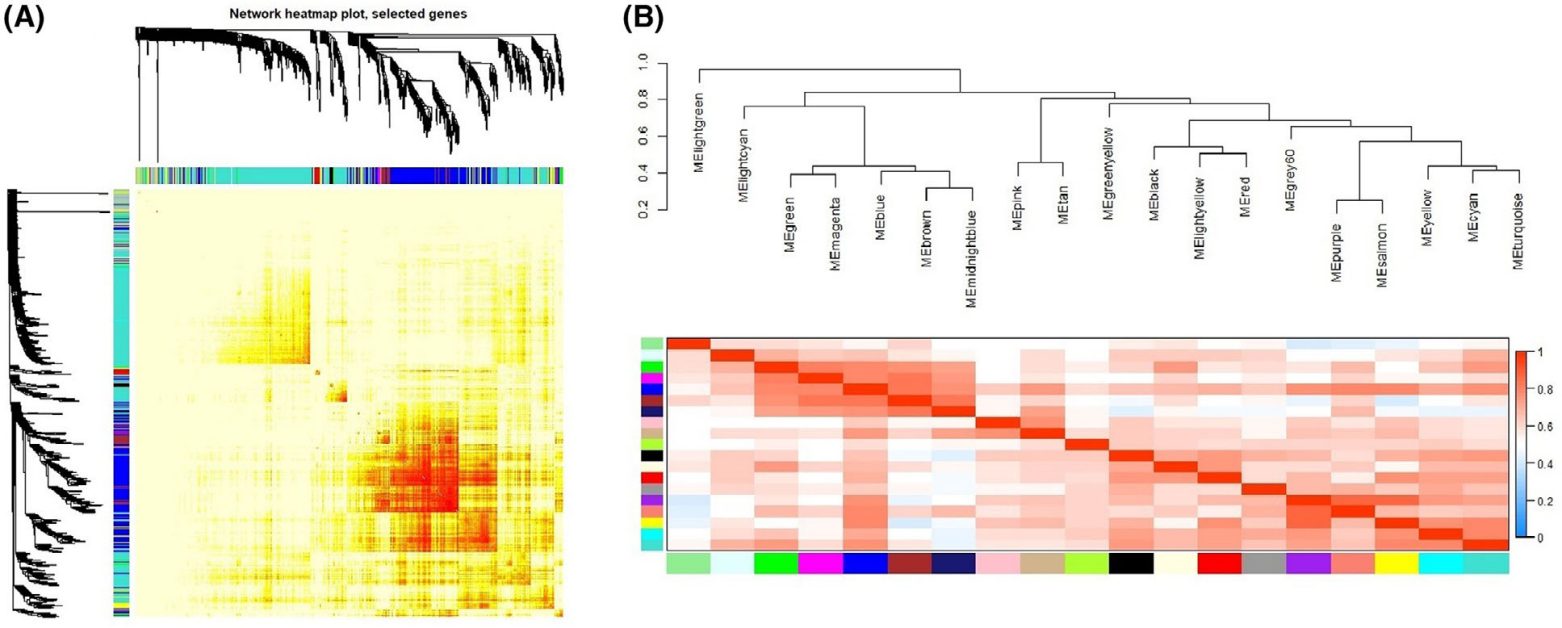
(A) A cluster visualization analysis was carried out on the expression of part of the mRNA. Different colors of horizontal axis and vertical axis represent different modules. There was no significant difference in interactions among different modules, indicating a high‐scale independence degree among these modules. (B) Hierarchical clustering of module hub genes that summarize the modules yielded in the clustering analysis.

The soft threshold of co‐expression modules of genes was selected to construct, and the powers were traversed from 1 to 30. It was found that when power = 7, the curves tended to be parallel and no longer converged. Therefore, power = 7 was selected for subsequent modules construction (Fig. 2). According to the mRNA expression of each group in GSE121212, cluster analysis was conducted (Fig. 3) and 20 modules of mRNA expression were obtained (Fig. 4). A cluster visualization analysis was carried out on the expression of part of mRNA (1000 genes were randomly selected for display) and modules, indicating that the genes under each module are independent of each other (Fig. 5A,B).

As shown in Figure 4, the MEgreen module has a significant positive correlation with the occurrence of AD, but a significantly negative correlation with the healthy sample, and a negative correlation with psoriasis, indicating that the gene under this module may be related to the pathogenesis of AD. The MEbrown module showed the highest positive correlation with the occurrence of psoriasis, but was negatively correlated with the healthy sample, and weakly associated with AD, indicating that the gene under this module may be related to the pathogenesis of psoriasis. Therefore, we identified the MEgreen module as the module most relevant to the AD, and defined the module most relevant to psoriasis as the MEbrown module.

### Function enrichment analysis

**Fig. 6:**
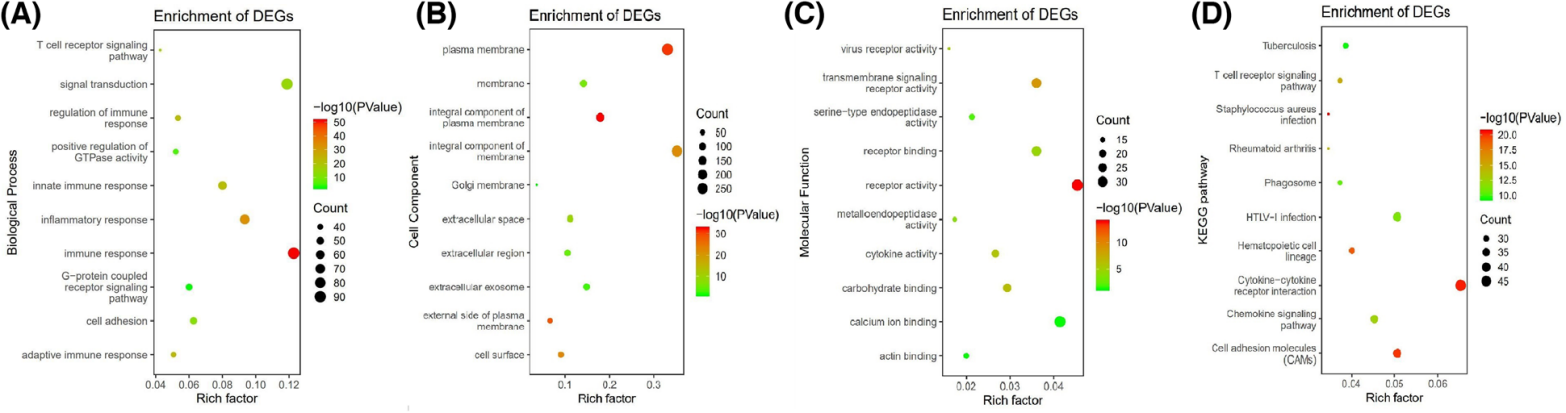
Functional and pathway enrichment analyses in the MEgreen module. (A) Biological process. (B) Cell component. (C) Molecular function. (D) KEGG pathways. The node size reflects the gene count, and the node color reflects the *P* value [−log_10_ (*P* value)].

**Fig. 7:**
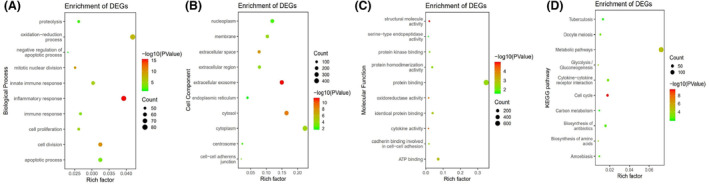
Functional and pathway enrichment analyses in the MEbrown module. (A) Biological process. (B) Cell component. (C) Molecular function. (D) KEGG pathways. The node size reflects the gene count, and the node color reflects the *P* value [−log_10_ (*P* value)].

The MEgreen and MEbrown module were assessed for further functional enrichment. GO enrichment of biological process was conducted using DAVID. GO categorization (biological process, cellular component and molecular function) of the MEgreen and MEbrown module are shown in Figs 6A–C and 7A–C. As the results show in the biological processes, the MEgreen module was mainly enriched in immune response, signal transduction, inflammatory response and innate immune response (Fig. 6A). In the MEbrown module, the results of enrichment analysis were mainly concerned with oxidation–reduction process, inflammatory response, apoptotic process and cell division (Fig. 7A).

Regarding the pathways in which these two modules were involved, we performed KEGG pathway analysis. The MEgreen module was enriched in pathways of cytokine–cytokine receptor interaction, cell adhesion molecules (CAMS) and human T cell leukemia virus type 1 (HTLV‐1) infection (Fig. 6D). In the MEbrown module, pathways concerned with metabolic pathway, cell cycle and cytokine–cytokine receptor interaction were significantly enriched (Fig. 7D).

### PPI network construction and hub genes identification

**Fig. 8:**
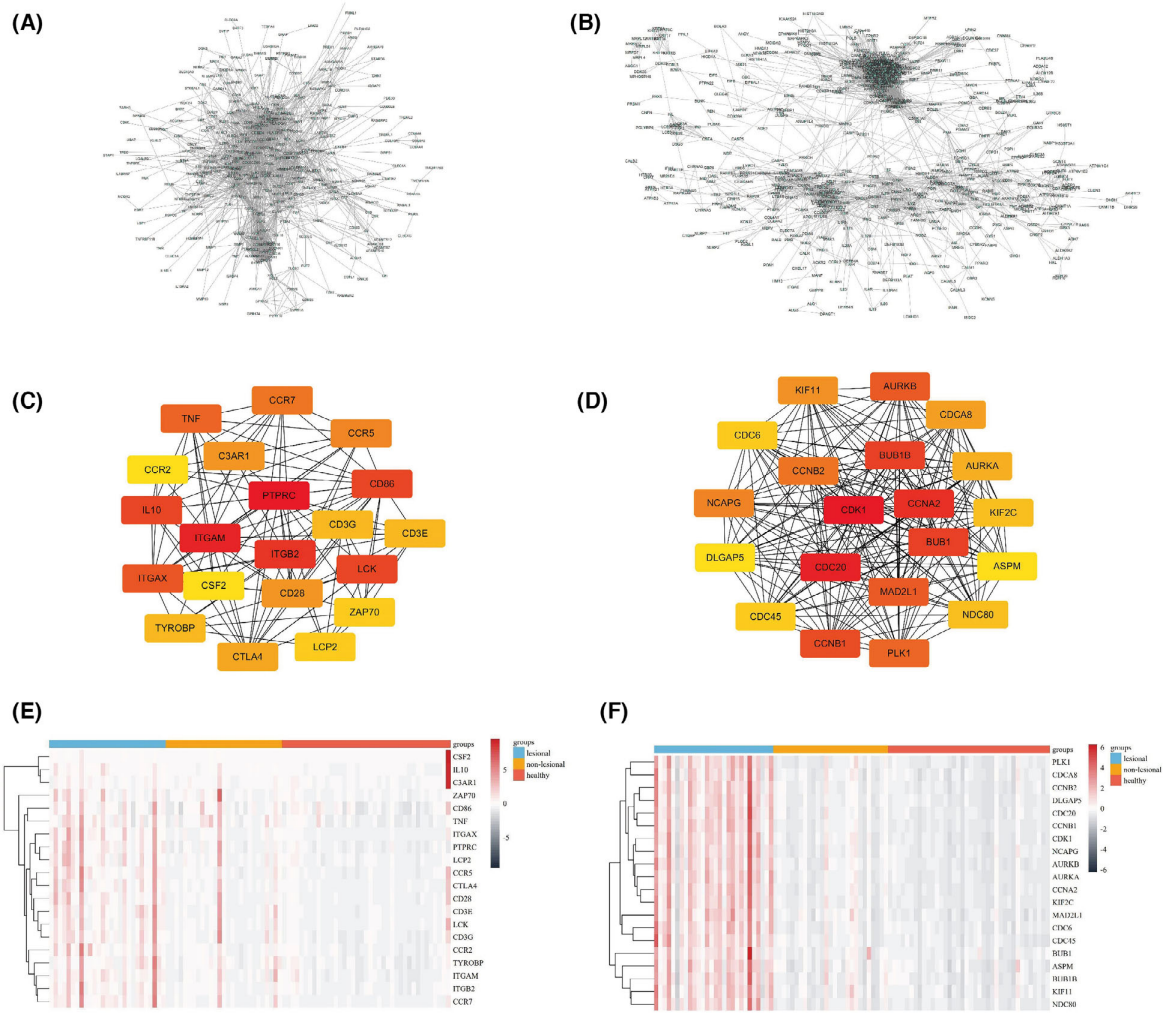
PPI and hub genes detection. (A) PPI network of genes in the MEgreen module. (B) PPI network of genes in the MEbrown module. (C) Selection of hub genes in the MEgreen module. (D) Selection of hub genes in the MEbrown module. (E) The expression of 20 hub genes associated with AD in GSE121212. (F) The expression of 20 hub genes associated with psoriasis in GSE121212.

We constructed a network of PPI for all genes in MEgreen and MEbrown module using cytoscape, consisting of 348 and 596 nodes according to the STRING database, respectively. The PPI network of MEgreen and MEbrown is presented in Fig. 8A,B. Moreover, We screened out the top 20 hub genes by sorting the node degree candidate gene. Figure 8C,D shows the top 20 hub genes in the MEgreen and MEbrown module. They were PTPRC, ITGAM, ITGB2, IL10, LCK, CD86, ITGAX, TNF, CCR7, CCR5, CD28, C3AR1, CTLA4, CD3E, TYROBP, CD3G, LCP2, ZAP70, CSF2 and CCR2 in the MEgreen module, and CDK1, CDC20, CCNA2, BUB1B, BUB1, CCNB1, MAD2L1, AURKB, PLK1, CCNB2, NCAPG, KIF11, CDCA8, AURKA, KIF2C, NDC80, CDC6, CDC45, ASPM and DLGAP5 in the MEbrown module. The expression level of the 20 hub genes associated with AD and psoriasis in GSE121212 is shown in Fig. 8E,F.

## Discussion

Although it has been confirmed that AD and psoriasis are inherited and mediated by the immune process, the exact pathogenesis of both skin diseases is not entirely understood. In the present study, WGCNA is used to construct co‐expression network of AD and psoriasis to predict clusters of genes involved in the pathogenesis of AD and psoriasis. We aimed to identify new and potential biomarkers that might contribute to the understanding of the molecular mechanism of AD and psoriasis, and the treatment of these two skin disease. GSE121212 was downloaded from the GEO database, and 20 co‐expression modules were obtained by WGCNA. Among the 20 modules, we found that the MEgreen and MEbrown module were most significantly related to AD or psoriasis respectively.

From our research, the MEgreen identified by WGCNA was the most significantly related module to AD and the MEbrown module was the most relevant module to psoriasis, indicating their importance in AD or psoriasis. We found that the MEgreen module played a role in immune response, signal transduction, inflammatory response and innate immune response. The KEGG pathway analysis revealed the MEgreen module to be mainly enriched in pathways of cytokine‐cytokine receptor interaction, CAMS and HTLV‐1 infection. The results were somewhat in accordance with those of previous research. Several studies have identified the important role of cell mediated immune responses in the pathophysiology of AD [6, 7, 8, 9, 10, 11]. Interestingly, we also found that HTLV‐1 infection is correlated with AD. HTLV is a human retrovirus that causes several diseases such as an aggressive form of leukemia. Recently, it has been reported that there are a further 14 diseases with significant associations or a substantially elevated risk with HTLV‐1, including eczema in children and asthma in males [12].

Regarding the MEbrown module, GO analysis revealed that it is involved in the oxidation–reduction process, inflammatory response, the apoptotic process and cell division. The results of KEGG pathway analysis showed that metabolic pathway, cell cycle and cytokine–cytokine receptor interaction were enriched. Because the pathogenic mechanism of psoriasis is considered to relate to the sustained inflammation that leads to uncontrolled keratinocyte proliferation and dysfunctional differentiation, we regard that the MEbrown module observed here as the important module in psoriasis pathogenesis [13]. In addition, the MEbrown module plays an important role in metabolism and may contribute to investigations of the mechanism of comorbid diseases of psoriasis such as metabolic syndrome [14].

Because of the importance of the MEgreen module in AD and MEbrown module in psoriasis, we screened the top 20 hub genes in these two modules. We found 20 hub genes PTPRC, ITGAM, ITGB2, IL10, LCK, CD86, ITGAX, TNF, CCR7, CCR5, CD28, C3AR1, CTLA4, CD3E, TYROBP, CD3G, LCP2, ZAP70, CSF2 and CCR2 in the MEgreen module associated with AD. In addition, we identified 20 hub genes CDK1, CDC20, CCNA2, BUB1B, BUB1, CCNB1, MAD2L1, AURKB, PLK1, CCNB2, NCAPG, KIF11, CDCA8, AURKA, KIF2C, NDC80, CDC6, CDC45, ASPM and DLGAP5 in the MEbrown module associated with psoriasis. Searching the GeneCards database and Open Targets platform, the 20 hub genes associated with AD are mainly involved in the process of immune system disease, cancer and infectious disease, and the 20 hub genes associated with psoriasis are mainly involved in the process of cell proliferation disorder and immune system disease. AD and psoriasis are autoinflammatory immune skin diseases, and the lesions of plaque psoriasis are manifested by excessive abnormal hyperplasia of the epidermis, suggesting that the hub genes are involved in the pathogenesis of AD and psoriasis.

## Conclusions

In summary, we have established a gene co‐expression network to identify network hub genes associated with the pathogenesis of AD or psoriasis by WGCNA. AD specific genes and psoriasis specific genes were found by bioinformatics analysis, which are conducive to exploring biomarkers for differential diagnosis or therapy. Recently, some studies have highlighted that AD and psoriasis may coexist in the same patient or develop consequently [15]. The mechanisms by which AD patients develop psoriasis or psoriatic patients develop AD are still not very clear, and some triggers such as biologics therapy can promote these processes [16]. Our research identified hub genes that help us better understand the pathogenesis of AD and psoriasis, and may aid in the development of certain biomarkers for diagnosing AD and psoriasis. Several limitations still exist in the present study. Further experimental researches are needed to confirm the role of these hub genes in the pathogenesis of AD and psoriasis, and appropriate biomarkers also need to be screened for use in clinical studies.

## Conflict of interest

The authors declare that they have no conflicts of interest.

### Peer review

The peer review history for this article is available at https://www.webofscience.com/api/gateway/wos/peer‐review/10.1002/2211‐5463.13686.

## Author contributions

YS and SZ conceived and designed the project. YS, SZ and JL acquired the data. YS and JL analyzed and interpreted the data. YS and SZ wrote the paper. All authors approved the final version of the manuscript submitted for publication.

## Data Availability

The data that support the findings of the present study are available from the corresponding author upon reasonable request.
